# Drug Resistance in *Toxoplasma gondii*

**DOI:** 10.3389/fmicb.2018.02587

**Published:** 2018-10-29

**Authors:** Mahbobeh Montazeri, Saeed Mehrzadi, Mehdi Sharif, Shahabeddin Sarvi, Asal Tanzifi, Sargis A. Aghayan, Ahmad Daryani

**Affiliations:** ^1^Toxoplasmosis Research Center, Mazandaran University of Medical Sciences, Sari, Iran; ^2^Student Research Committee, Mazandaran University of Medical Sciences, Sari, Iran; ^3^Razi Drug Research Center, Iran University of Medical Sciences, Tehran, Iran; ^4^Department of Parasitology, School of Medicine, Sari Branch, Islamic Azad University, Sari, Iran; ^5^Department of Parasitology, Sari Medical School, Mazandaran University of Medical Sciences, Sari, Iran; ^6^Laboratory of Zoology, Research Institute of Biology, Yerevan State University, Yerevan, Armenia

**Keywords:** *Toxoplasma gondii*, toxoplasmosis, drugs, drug resistance, mechanisms of resistance

## Abstract

*Toxoplasma gondii* (*T. gondii*) is a global protozoan parasite infecting up to one-third of the world population. Pyrimethamine (PYR) and sulfadiazine (SDZ) are the most widely used drugs for treatment of toxoplasmosis; however, several failure cases have been recorded as well; suggesting the existence of drug resistant strains. This review aims to give a systematic and comprehensive understanding of drug resistance in *T. gondii* including mechanisms of resistance and sites of drug action in parasite. Analogous amino acid substitutions in the *Toxoplasma* enzyme were identified to confer PYR resistance. Moreover, resistance to clindamycin, spiramycin, and azithromycin is encoded in the rRNA genes of *T. gondii*. However, *T. gondii* SDZ resistance mechanism has not been proved yet. Recently there has been a slight increase in SDZ resistance. That is why the majority of studies were carried out using SDZ. Six strains resistant to SDZ were found in clinical cases between 2013 and 2017 which among Brazilian *T. gondii* isolates, TgCTBr11, Ck3, and Pg1 were identified in human toxoplasmosis, as well as in livestock intended for human consumption. In conclusion, recent experimental studies in clinical cases have clearly shown that drug resistance in *Toxoplasma* is ongoing. Thus, establishing a more effective therapeutic scheme in the treatment of toxoplasmosis is critically needed. The emergence of *T. gondii* strains resistant to current drugs, reviewed here, represents a concern not only for treatment failure but also for increased clinical severity in immunocompromised patients. To improve the therapeutic outcome in patients, a greater understanding of the exact mechanisms of drug resistance in *T. gondii* should be developed. Thus, monitoring the presence of resistant parasites, in food products, would seem a prudent public health program.

## Introduction

*Toxoplasma gondii* (*T. gondii*) is a remarkably successful protozoan parasite that belongs to the phylum Apicomplexa (Tenter et al., 2000). It is estimated that up to one-third of the world's human population is infected with *T. gondii* (Montoya and Liesenfeld, 2004). In addition, toxoplasmosis is considered as the third most common food-borne parasitic infection requiring hospitalization (Vaillant et al., 2005).

*Toxoplasma* infection in humans mainly occurs through two ways: (1) ingestion of tissue cysts with raw or undercooked meat. (2) Consumption of oocysts with contaminated food, water, vegetables, fruits, etc. Congenital transmission from mother to fetus is also possible when a woman gets an infection during pregnancy (Moncada and Montoya, 2012; Sepúlveda-Arias et al., 2014).

In most immunocompetent people, infection with *T. gondii* is usually asymptomatic. But in immunocompromised or congenitally infected patients without proper treatment, severe diseases may occur (Moncada and Montoya, 2012; Wang et al., 2017). In a recent global meta-analysis study, high odds ratios (ORs) was reported for *Toxoplasma* infection in HIV/AIDS patients especially in Asia and Africa and in cancer patients in Asia (Wang et al., 2017). However, in South America, severe ocular toxoplasmosis is higher than in many other parts of the world (Glasner et al., 1992).

The population structure of *T. gondii* consists of three main clonal lineages; Type I (including a highly virulent RH strain), Type II (including ME49 and PRU, avirulent strains), and Type III (including avirulent strains like NED) (Howe and Sibley, 1995).

Type II is the predominant type of clonal lineage that infects humans and animals in Europe and in North America. However, more recent studies in South America have documented the discovery of genetically atypical (non-clonal) strains of *T. gondii* isolated from human patients, which caused much more dramatic clinical symptoms compared with their European counterparts. Thus, the number of strains, or isolates, not to be categorized as type I, II, and III clonal lineages has increased strongly, and has meanwhile outnumbered those who are conventionally categorized (Shwab et al., 2014). These atypical strains also influenced immunocompetent individuals, and there are reports of abortions in *Toxoplasma*-positive pregnant women due to atypical *T. gondii* strains. Phenotypically, atypical *T. gondii* field strains are completely different from their European counterparts and from laboratory-adapted strains used as models for studies on *T. gondii* biology and the efficacy of novel compounds in drug development programs (Shwab et al., 2014).

Recommended drugs for treatment or prophylaxis of toxoplasmosis are limited to combinations of pyrimethamine (PYR) and sulfadiazine (SDZ). Unfortunately, these drugs have severe side effects such as neutropenia, leucopenia, severe platelet count decrease, thrombocytopenia, and hypersensitivity reactions (Porter and Sande, 1992; Rajapakse et al., 2013; Montazeri et al., 2015). Additionally, these drugs are related to some uncommon reactions as well, including agranulocytosis, Stevens–Johnson syndrome, toxic epidermal necrolysis, and hepatic necrosis, which may be fatal in patients with toxoplasmosis (McLeod et al., 2006).

In a retrospective review, 62% of patients treated with PYR, SDZ, and leucovorin showed a high rate of toxication and a number of side effects which required a change in the therapeutic regimen in 44% of patients (Porter and Sande, 1992).

Drugs, such as azithromycin, clarithromycin, spiramycin, atovaquone, dapsone, and cotrimoxazole (trimethoprim-sulfamethoxazole), have also been used to treat clinical toxoplasmosis; however, they are poorly tolerated and have no effect on the bradyzoite form of the parasite (Montazeri et al., 2017b, 2018).

Also, there have been several reports on failures of the long-term treatment of toxoplasmic encephalitis, chorioretinitis, and congenital toxoplasmosis with antifolate, particularly among AIDS patients (Jacobson et al., 1996; Bossi et al., 1998; Villena et al., 1998. Hence, there is controversy whether these failures are related to pharmacological parameters (drug intolerance, poor compliance, and malabsorption) and/or to the development of drug-resistant parasites or a lower susceptibility of the *T. gondii* strain (Meneceur et al., 2008). *T. gondii* parasite has an exceptional adaptive potential which renders it “resistant,” but the mechanism of resistance, or adaption, has not been completely elucidated (Kropf et al., 2012).

In a previous study by Ouellette, the basic mechanisms of parasite drug resistance in malaria, leishmaniasis, sleeping sickness, and common helminthiases were evaluated (Ouellette, 2001). In another study by McFadden et al. resistance was investigated as a tool to investigate old and new drug action sites in *Toxoplasma* parasite (McFadden et al., 2001). However, previous studies have demonstrated that drug resistance in *T. gondii* is not yet a major problem in human population (McFadden et al., 2001; Sims, 2009); recently, studies have focused on finding safe drugs with novel mechanisms of action for toxoplasmosis that are both efficacious and nontoxic for patients (Alday and Doggett, 2017; Montazeri et al., 2017a; Daryani et al., 2018).

It should be noted that various studies have been published reporting drug resistance in *T. gondii* (Table 1). The fact that drug resistant forms of *T. gondii* strains can contribute to human disease could raise a concern for treatment failure in the future (Silva et al., 2017). This review is focused on the available knowledge, encompassing information on anti-*Toxoplasma* drug resistance including mechanisms of resistance and drug target in parasite.

**Table 1:** List of the studies that evaluated drug resistance in *T. gondii*.

**No**	**Drug**	**Treatment (dose/route/and time)**	***In vitro/in vivo***	**Acute/Chronic**	**Host/Cell line**	**Strain**	**Resistant strain**	**Focus of the study**	**Method**	**Main results**	**Resistance mechanism**	**References**
**1**	Anticoccidial drugs	0.001–100 μg/ml	*In vitro*	Acute	HFF	RH	–	Resistance development *in vitro*	Incorporation of [^3^H]uracil and plaque assays	The resistance in *T. gondii* by attempting to select mutants *in vitro* from parasites mutagenized with ethylnitrosourea was explored	–	Ricketts and Pfefferkorn, 1993
**2**	Arprinocid and arprinocid-N-oxide	360 μg/orally	*In vitro/in vivo*	Acute	Swiss mice/HFF	RH	R-Ano^R^-1	A mutant resistant to arprinocid-N-oxide	Incorporation of [^3^H]uracil and plaque assays/survival rates	A parasite mutant, R-Ano^R^-1 was isolated that was 16–20-fold more resistant to arprinocid-N-oxide than was the wild type RH *T. gondii*. This mutant was not resistant to arprinocid *in vitro*	–	Pfefferkorn et al., 1988
**3**	Artemisinin	35 μg/mL	*In vitro*	Acute	HFF	RH	–	The mechanism of action	Incorporation of [^3^H]uracil	Mutants resistant were selected to better understand its inhibitory effects on *T. gondii*	–	Berens et al., 1998
**4**	Artemisinin	2.4, 12, or 300 μg/ml	*In vitro*	Acute	HFF	RH, clone 2F	KN200-1, KN200-6. and STL500-10A	The molecular mode of action	Microneme secretion assays, calcium monitoring, sequencing, and qRT-PCR	Calcium homeostasis is the mechanism of action of artemisinins against apicomplexan parasites	Altering in calcium homeostasis	Nagamune et al., 2007
**5**	Atovaquone	1 μM	*In vitro*	Acute	HFF	ME49	R4, R5, R7, and R32	The mechanisms of resistance	Incorporation of [^3^H]uracil, RT-PCR^a^ and northern blot analysis	Atovaquone interfered with electron transport at the cytochrome *bc*1 complex in *T*. *gondii*	Q_o_ domain of cytochrome b	McFadden et al., 2000
**6**	Atovaquone	25 nM	*In vitro*	Acute	Non-fermentable medium	RH	M129L and I254L	Molecular basis of resistance	Measurement of oxygen consumption, ubiquinol-cytochrome c reductase activity and molecular modeling	With the two mutations from *T. gondii*, M129L and I254L, we have a database of 13 point mutations surrounding the atovaquone binding site	A hydrophobic region of the binding pocket of the cytochrome bc_1_ complex	Kessl et al., 2006
**7**	Atovaquone (566C80) or decoquinate	0.1 and 0.01 μM	*In vitro*	Acute	HFF	RH	Deq^R^-1 and Ato^R^-1	The mechanisms of resistance	Incorporation of [^3^H]uracil, plaque assays, and oxygen uptake	*De novo* pyrimidine synthesis was not the major biochemical target of atovaquone and decoquinate	–	Pfefferkorn et al., 1993
**8**	Atovaquone and SDZ^b^	6.25, 12.5, 25, 50/40, 80, 160, and 320 mg/kg/day/10 days/gavage	*In vivo*	Acute/Chronic	Swiss Webster mice	RH, SAF, EGS, D4, D7, CH1, and CH3	–	The efficacy of drugs for the treatment of mice infected with six strains isolated in Minas Gerais, Brazil	PCR-RFLP, survival rates, the presence of brain cysts, ELISA^c^, and bioassay	Type I strains was more resistant to atovaquone	Alterations in genes encoding these drugs targets	Alves and Vitor, 2005
**9**	Azithromycin, spiramycin, or clindamycin	100 ng/ml	*In vitro*	Acute	HFF	RH	Cln^R^-2, Azi^R^-l, or Spr^R^-1	The mechanism of action	Incorporation of [^3^H]uracil, plaque assay, and mitochondrial function measured by oxygen uptake	Mitochondrial protein synthesis was not the target of clindamycin or azithromycin	rRNA genes of the 35-kb genome	Pfefferkorn and Borotz, 1994a
**10**	Clindamycin	Up to 100 μg/ml	*In vitro*	Acute	HFF	RH	Cln^R^-2	The mechanism of action	Incorporation of [^3^H]uracil and plaque assays	A difference between the wild type and Cln^R^-2 in a mitochondrial ribosomal protein or in methylation of mitochondrial rRNA was seen	A mitochondrial ribosomal protein or mitochondrial rRNA genes	Pfefferkorn et al., 1992b
**11**	Diclazuril	0.0025, 0.005, 0.01, 0.1, and 1.0 μg/ml	*In vitro/in vivo*	Acute	HFF/Mice	RH, 2 tissue cyst formers, GT-1, and WTD-3	Dic^R^-1	A resistant mutant	Transmission electron microscopy assays/survival rates and cysts count in mice brains	Dic^R^-1 mutant of the RH strain, resistant to 1.0 μg/ml diclazuril	–	Lindsay et al., 1995
**12**	Fosmidomycin	100 mg/kg/10 days	*In vitro/in vivo*	Acute	HFF/Webster mice	RH	–	The mechanisms of resistance	PCR, sequencing, immunofluorescence, and western blotting assays	*Toxoplasma* DOXP^d^ pathway is essential in parasites that are highly fosmidomycin resistant	Target DOXP reductoisomerase	Nair et al., 2011
**13**	FUDR^e^	20 μg/ml	*In vitro*	Acute	HFF	RH	FUDR^R^-1	The mechanisms of resistance	Plaque assays, autoradiography, and a modified Schmidt-Thannhauser fractionation	The FUDR-resistant was resistant to wildtype *T. gondii*, fluorouracil, and fluorouridine	Pyrimidine salvage pathways	Pfefferkorn and Pfefferkorn, 1977
**14**	5-FUDR and araA^f^	20 μg/ml	*In vitro/in vivo*	Acute/Chronic	Mice/HFF	C strain	FR5, C-FUDR^R^-2 and C-ara-A^R^-l	Genetic recombination with *T. gondii*	Immunofluorescence, plaque, enzyme, isotopic, and spectrophotometrically protein assays/cysts count in mice brains	Genetic recombination can readily be demonstrated with suitable mutants of *T. gondii*	–	Pfefferkorn and Pfefferkorn, 1980
**15**	FUDR, HU^g^, araA, and SF^h^	FUDR, 10^−5^; HU, 2.4 × l0^−4^, araA, 3 × 10^−4^, SF, 2.7 × 10^−7^ M	*In vitro/in vivo*	Acute/Chronic	Mice/HFF	C strain	C-FUDRR-2, C-HIJR-1, C-ara-AR-l, and C-SFR-1	Genetic recombination between two different drug-resistant mutants of *T. gondii*	Plaque assays/cysts count in mice brains	The gene for FUDR resistance phenotypically suppressed the gene for HU resistance	–	Pfefferkorn and Kasper, 1983
**16**	1-Hydroxyquinolones	10–100 nM	*In vitro*	Acute	HFF	RH	N302S TgDHODH^d^	*T. gondii* TgDHODH as a relevant HDQ^i^ target	PCR, sequencing, plasmid cloning, cDNA synthesis, replication assay, and Enzyme kinetics	The mode of action of HDQ on the *T. gondii* physiology appears to be a combination of the inhibitionof energy metabolism and an inhibition of *de novo* pyrimidine synthesis	Restoration of *de novo* pyrimidine biosynthesis	Hegewald et al., 2013
**17**	Monensin	2 ng/ml/24 h	*In vitro*	Acute	HFF	RH strain lacking a functional hpt gene	–	Isolation of a *T. gondii* mutant resistant to monensin and the drug-resistant phenotype	Plaque assays, PCR, cloning of TgMSH-1^j^, southern blot, and immunofluorescence assays	Disruption of TgMSH-1, an MSH in *T. gondii*, confers drug resistance	Disruption of mitochondrion TgMSH-1	Garrison and Arrizabalaga, 2009
**18**	1NM-PP1	250 or 1,000 nM/3 weeks	*In vitro*	Acute	Vero cells	PLK/DUAL and PLK/hxgprt^_^	PLK/DUAL res.1 and PLK/DUAL res.2	The mechanism of resistance to 1NM-PP1	PCR, sequencing, invasion, cell division, calcium-induced egress, and plaque assays	TgMAPK1 as a novel target for 1NM-PP1 activity	The mutation in TgMAPK1	Sugi et al., 2013
**19**	Oryzalin	0.5 or 2.5 μM	*In vitro*	Acute	HFF	RH	49 independent resistant *T. gondii* lines	The mechanisms of resistance	PCR and sequencing	*Toxoplasma* resistance to oryzalin is associated with point mutations to α-1-tubulin	α-1-tubulin	Morrissette et al., 2004
**20**	Oryzalin	0.5 or 2.5 μM	*In vitro*	Acute	HFF	RH	–	Identification of resistance mutations confer resistance in *Toxoplasma*	PCR, sequencing immunofluorescence staining and flow cytometry	Mutations to α-1-tubulin confer dinitroaniline resistance at a cost to microtubule function and *T. gondii* fitness	α-1-tubulin	Ma et al., 2007
**21**	Oryzalin	0.5 μm	*In vitro*	Acute	HFF	RH	46 resistant *T. gondii* lines	The development of new anti-parasitic therapies	PCR, sequencing immunofluorescence staining, and flow cytometry	46 *T. gondii* lines were isolated that have suppressed microtubule defects associated with the G142S or the F52Y mutations by acquiring secondary mutations	α-1-tubulin	Ma et al., 2008
**22**	PYR^k^	1 μM	*In vitro*	Acute	HFF	RH	M2, M3, M4, M2M3, M2M4, and M3M4	The mechanisms of resistance	Incorporation of [^3^H]uracil, plaque assays, PCR, and sequencing	Analogous amino acid substitutions have identified in the *Toxoplasma* enzyme that confer drug resistance to transfected parasites	Analogous amino acid substitutions in amino acid	Donald and Roos, 1993
**23**	PYR	0, 5, 10, 15, or 20 μM	*In vitro*	Acute	HFF	RH, P(lK), and veg	W25R, L98S, and l134H	The potential role of *dhfr*^l^polymorphisms	Incorporation of [^3^H]uracil, PCR, and sequencing	PYR is a potent inhibitor of DHFR and three resistance mutations were identified, at amino acid residues	Analogous amino acid substitutions in amino acid residues	Reynolds et al., 2001
**24**	PYR, atovaquone, and SDZ	0.002–1/0.001–0.5/0.0005–100 mg/l/gavage	*In vitro, in vivo*	Chronic	MRC-5, THP-1 cells/White rabbit	RH, B1, ENT, ME49, and 10 strains from patients with congenital toxoplasmosis^m^	B1, RMS-1995-ABE, and RMS-2001-MAU	The susceptibilities of *T. gondii* strains belonging to various genotypes to drugs	Specific enzyme-linked immunosorbent assay, qRT-PCR, PCR, and direct sequencing	A higher variability was found for SDZ, with a possible resistance of three strains	–	Meneceur et al., 2008
**25**	PYR, 5-fluorouracil, and 5-fluorocytosine	1 μM	*In vitro*	Acute	HFF	RH	–	The development of improved model genetic systems	DNA extraction, [^3^H]Xanthine incorporation, southern blot, and western blot analysis	Exogenously supplied cytosine or uracil rescued the growth of CD transgenic *T. gondii* that were cultured in the presence of cytotoxic concentrations of pyrimidine compounds	DHFR-TS^n^ gene	Fox et al., 1999
**26**	SDZ	0–10 mM	*In vitro*	Acute	Mice/Tissue culture	RH	R-Sul^R^-5 and Swa-20	Identification of SDZ-resistant strains of *T. gondii* in likely sources of human infection	PCR and sequencing, expression, and purification of protein	The human-derived allelic form encoding the SDZ-resistant enzyme was found in *T. gondii* associated with a fatal infection	Amino acid residues corresponding to DHPS-407	Aspinall et al., 2002
**27**	SDZ	0, 75, and 1,000 μg/mL/72 h	*In vitro*	Acute	Vero cells	RH and ME49	RH-^R^SDZ, ME-49-^R^SDZ, TgA 103001, TgH 32006, and TgH 32045	Identification of genotypic and/or phenotypic markers of SDZ resistance	PCR, qRT-PCR, and nucleotide sequence	*T. gondii* SDZ resistance is not related to three ABC genes, TgABC.B1, TgABC.B2, and TgABC.C1	–	Doliwa et al., 2013a
**28**	SDZ	0, 75, and 1,000 μg/mL/72 h	*In vitro*	Acute	Vero cells	RH and ME49	RH-^R^SDZ, ME-49-^R^SDZ, TgA 103001 and TgH 32006	The development of two SDZ-resistant strains	ELISA and enzyme immunoassay	IC_50_-values of SDZ were higher than 1,000μg/mL for the two natural resistant strains (RH-R^SDZ^ and ME-49-R^SDZ^)	–	Doliwa et al., 2013a
**29**	SDZ	0, 75, and 1,000 μg/mL/72 h	*In vitro*	Acute	Vero cells	RH and ME49	TgA 103001, TgH 32006, and TgH 32045	The mechanisms of resistance	Q-RT-PCR, western blot, Real-time qRT-PCR, DIGE^o^, sypro ruby staining, and mass spectrometry analyses	SDZ resistance in *T. gondii* resistant strains was isolated from clinical cases	Differentially expressed proteins	Doliwa et al., 2013c
**30**	SDZ	0.2–2 μM/ip^p^	*In vitro/in vivo*	Acute	Swiss white mice/HFF	RH	R-Sul^R^-5	SDZ resistance in R-Sul^R^-5 mutant of *T. gondii*	Incorporation of [^3^H]uracil and plaque assays/ survival rates	R-Sul^R^-5 was resistant to SDZ *in vitro* and *in vivo*	Inhibit the synthesis of dihydropteroic acid and, the synthesis of dihydrofolic acid	Pfefferkorn et al., 1992a
**31**	SDZ	500 mg/L/orally/10 days/l,100, 200, or 300 mg/kg/ip/6 days	*In vivo*	Acute/Chronic	Swiss mice	RH and ME49	TgCkBrRN3 (Ck3) and TgPgBrRN1 (Pg1)	Identification of the pathogenicity and phenotypic SDZ resistance	Parasite isolation/survival rates, ELISA, PCR-RFLP, PCR, and sequencing	The Ck3 and Pg1 isolates showed SDZ resistance	–	Oliveira et al., 2016
**32**	SDZ	80, 160, or 320 mg/Kg/day/gavage/10 days	*In vivo*	Acute	Swiss mice	RH, GTI, ME49, VEG, TgCTBr03, 07, 08, 11, and 16	TgCTBr11	Identification of polymorphisms and profile of resistance to SDZ	PCR-RFLP, survival rates, cyst count, and ELISA assay	TgCTBr11 isolate presented a profile of resistance to SDZ	–	Silva et al., 2017
**33**	SDZ, atovaquone, clindamycin, rotenone, antimycin, myxothiazol, and adenosine arabinoside	0.01, 0.1, and 0.5 mM/1.5, 50, and 150 nM/0.1, 1, and 5 μg/ml/2, 20, and 100 μM	*In vitro*	Acute	HFF or Vero cells	PLK	–	The interconversion of tachyzoite to bradyzoite	Incorporation of [^3^H]uracil, SDS-polyacrylamide gel electrophoresis, and western blots	The drugs targeted to mitochondria will cause wild type parasites to differentiate from tachyzoites to bradyzoites	–	Tomavo and Boothroyd, 1995
**34**	6-Thioxanthine	20, 40, and 360 μg/m	*In vitro*	Acute	HFF	RH	Thx^R^-1	The mechanisms of resistance	Incorporation of [^3^H]thymidine	The lack of the hypoxanthine-guanine phosphoribosyl transferase is the basis for the resistance of Thx^R^-1–6-thioxanthine	The lack of the enzyme to 6-thioxanthine	Pfefferkorn and Borotz, 1994a

a Reverse transcription polymerase chain reaction.

b Sulfadiazine.

c Enzyme-linked immunosorbent assay.

d 1-deoxy-d-xylulose-5-phosphate.

e Fluorodeoxyuridine.

f Adenine arabinoside.

g Hydroxyurea.

h Sinefungin.

i 1-hydroxy-2-dodecyl-4(1) quinolone.

j T. gondii MutS homologs.

k Pyrimethamine.

l Dihydrofolate reductase.

m RMS-1995-ABE, TRS-2004-REV, TOU-1998-TRI, RMS-2005-HAG,GRE-1995-MAE,PSP-2005-MUP,GRE-1998-TRA, RMS-2003-TOU, NED, RMS-1994-LEF, RMS-2003-DJO, RMS-2001- MAU, GUY-2003-MEL.

n Dihydrofolate reductase-thymidylate synthase.

o Difference-gel electrophoresis.

p Intraperitoneally.

## Pyrimethamine resistance

Clinically, acute toxoplasmosis is usually treated with a combination of PYR and SDZ. These drugs inhibit important enzymes for pyrimidine biosynthesis in the parasite [dihydrofolate reductase (DHFR) and dihydropteroate synthase (DHPS)] and have a remarkable synergistic activity against parasite survival and replication. DHFR is also present in humans so that the treatment with DHFR inhibitors may induce a folate deficiency state, which is probably responsible for hematological side effects and embryopathies (Rajapakse et al., 2013). Therefore, in order to limit adverse hematological events, these treatments are administered with folinic acid (Alday and Doggett, 2017).

However, these pathways are not essential for *T. gondii* viability, as studies showed that in resistant strains of *T. gondii* to 5-fluorodeoxyuridine, adenosine arabinoside, and 6-thioxanthine, the purine and pyrimidine analogs, isolated *in vitro* were viable despite defects in the crucial nucleotide synthesis enzymes (Pfefferkorn and Pfefferkorn, 1977, 1978; Pfefferkorn and Borotz, 1994b).

Reynolds et al. (2001) reported that using *in vitro* mutagenesis, single-point mutations in *T. gondii* DHFR-TS (dihydrofolate reductase-thymidylate synthase) (e.g., W25R, L98S, and L134H) can produce drug resistance in RH strain parasites compared with type II and type III strains. In addition, using site directed mutagenesis and transgenic experiments several mutations were induced in the DHFR-TS gene related to resistance to PYR. The T83N mutation was found to probably confer resistance to PYR (Donald and Roos, 1993). Resistance is even increased when T83N mutation is associated with mutation of S36R and F245S (Reynolds et al., 2001). Meneceur et al. reported variability in the susceptibilities of *T. gondii* strains to PYR, with no clear evidence of drug resistance and no relationship with strain genotype or defined mutations in drug target genes (Meneceur et al., 2008).

Given that PYR resistance is differently marked among various strains of *T. gondii*; it can provide new insights into potential sources of treatment failures and possible drug resistance mechanisms.

## Sulfonamides resistance

Sulfonamides, in conjunction with PYR, are a mainstay of toxoplasmosis treatment, although AIDS patients are unable to tolerate this treatment. The first experimentally induced drug-resistance was resistance to sulfamethoxazole, when the parasite was exposed to sub-lethal doses of the drug for long periods (Sander and Midtvedt, 1971; Luft and Remington, 1992; Reynolds and Roos, 1998). In a study by Pfefferkorn et al. (1992a), researchers induced resistance in RH strain using chemical mutagenesis and growing parasites in environments with gradually increased SDZ concentrations. Sulfamethoxazole-resistant strain (R-Sul^R^-5) appeared to be more resistant than the parental RH strain. Further study on R-Sul^R^-5 confirmed previous findings that this strain is sulfonamide resistant with an IC_50_ value near 5 mM like another SDZ resistant strain Swa-20 which was isolated from patients with clinical toxoplasmosis (Aspinall et al., 2002. In Aspinall et al. (Aspinall et al., 2002) study, the presence of one mutation at positions 407 of DHPS was associated with sulfonamides resistance by direct sequencing of PCR products (Aspinall et al., 2002). This mutation was also retrieved in laboratory induced R-Sul^R^-5 (Pfefferkorn et al., 1992a). As, mutation 407 was not identified in five *T*. *gondii* Brazilian isolates obtained from newborns with congenital toxoplasmosis (Silva et al., 2017), a larger number of atypical isolates of *T. gondii* must be evaluated to confirm these results.

Meneceur et al. (Meneceur et al., 2008) isolated three strains from clinical cases containing: TgA 103001, previously described as B1 (Type I strain), TgH 32006, previously described as RMS-1995-ABE (Type II strain), and TgH 32045, previously described as RMS-2001-MAU (Type II variant strain) which were detected as resistant to SDZ. Doliwa et al. (2013c) found 44% over-expressed proteins in resistant *T. gondii* strains. The virulence-associated rhoptry protein, ROP2A, was found in greater abundance in both naturally resistant Type II strains TgH 32006 and TgH 32045. Totally, 31 proteins were identified which are differentially modulated between SDZ resistant and sensitive strains of *T. gondii* according to their genotype using proteomics approach. Recently, two SDZ resistant strains were developed called RH-R^SDZ^ and ME-49-R^SDZ^ *in vitro* (Doliwa et al., 2013b). Also, other studies analyzed genotypic and/or phenotypic markers of resistance in *T. gondii* (Doliwa et al., 2013a).

In Oliveira et al. (Oliveira et al., 2016) study, Ck3 and Pg1 *T. gondii* isolates showed SDZ resistance in samples collected from livestock intended for human consumption. Monitoring the presence of resistant parasites, particularly in food products, would seem a prudent public health measure (Sims, 2009).

Silva et al. (2017) have confirmed the existence of a Brazilian *T. gondii* isolate, TgCTBr11, isolated from newborns infected with congenital toxoplasmosis, which is resistant to SDZ. Despite the large number of polymorphisms identified in the DHPS gene, no association was found between the profile of susceptibility to SDZ and the virulence-phenotype and genotype of the parasite. However, the mutation in the DHPS gene is known to confer resistance in *T. gondii* and has demonstrated cross-resistance to several sulfonamides including SDZ and sulfamethoxazole. Until now, *T. gondii* SDZ resistance has not been related to genetic mutations in DHPS in all clinical isolates. Based on these findings, the range of resistance to sulfonamide is greater than PYR or atovaquone.

## Atovaquone resistance

Atovaquone is a substituted hydroxynaphthoquinone compound that is being used clinically for the treatment of *T. gondii* infections against chronic bradyzoite stage via mitochondrial electron transport chain inhibition (Kovacs, 1992; Tomavo and Boothroyd, 1995); however, atovaquone prophylaxis and treatment failure was reported in hematopoietic cell transplant recipients and AIDS patients (Chirgwin et al., 2002; Gajurel et al., 2016).

Cytochrome bc_1_ complex (CYT bc_1_) is a membrane-bound enzyme of the respiratory electron transfer chain located in the inner mitochondrial membrane. It is a successful drug target for combatting diseases, including *T. gondii, Plasmodium falciparum*, and *Babesia microti* (Winter et al., 2008; Doggett et al., 2012; Lawres et al., 2016). CYT bc_1_ reduces cytochrome c and generates an electrochemical gradient by transferring protons to the intermembrane space. It also creates ubiquinone for pyrimidine biosynthesis. CYT bc_1_ has two active sites, the bc_1_ Qo site (oxidizes ubiquinol) and the bc_1_ Qi site (reduces ubiquinone) (Crofts, 2004).

The genetic evidence revealed that atovaquone, targets *T. gondii* CYT bc_1_ by binding to Q_o_ domain of cytochrome *b* confer resistance to atovaquone (McFadden et al., 1997, 2001). M129L and I254L mutations have been identified to be related to atovaquone resistance in *T. gondii* (31, 32).

Of course, the investigation by Meneceur et al. (Meneceur et al., 2008) did not show any of these mutations, thus further studies will help a better understanding of resistance mechanisms.

Interestingly, Endochin-like quinolones (ELQs) have been shown to be active against atovaquone-resistant *Plasmodium* and *Babesia* (Winter et al., 2008; Lawres et al., 2016). Also, treatment with 4(1H)-pyridone compounds, GW844520, and GSK932121, showed anti-malarial activity *in vivo* (Capper et al., 2015). These anti-parasitic agents have a similar mechanism of action with atovaquone but by inhibiting the Qi site of CYT bc_1_. Furthermore, ELQ-271 and ELQ-316 showed remarkable effects against acute and latent toxoplasmosis at low doses (Doggett et al., 2012). It is likely that ELQs act at the *T. gondii* cytochrome b Qi site. Therefore, ELQs and 4(1H)-pyridone compounds are promising candidates for the treatment against atovaquone-resistant *Toxoplasma*.

## Mutants of *T. gondii* resistant to 1-Hydroxyquinolones

1-Hydroxyquinolones are effective inhibitors of *T. gondii* replication. Using a drug resistant strain, Hegewald et al. (2013) described that the enzyme dihydroorotate dehydrogenase (TgDHODH) of *T. gondii* is a relevant target for 1-Hydroxy-2-dodecyl-4(1*H*) quinolone (HDQ) and compound B (1-Hydroxyquinolones derivatives). Thus, drug resistant mutants are approved tools for the identification of drug targets for future to select new anti-*Toxoplasma* drugs.

## Mutants of *T. gondii* resistant to clindamycin, spiramycin, and azithromycin

Antibiotics such as clindamycin, spiramycin, and azithromycin are known to be active against *T. gondii*. However, mutant Cln^R^-2 (RH) was cross-resistant to clindamycin, azithromycin, and spiramycin antibiotics (Pfefferkorn et al., 1992b; Pfefferkorn and Borotz, 1994a). Interestingly, resistance to these drugs is encoded in the rRNA genes of the 35-kb genome in *T. gondii* and the apicoplast protein synthesis is known as target of these antibiotics action against *T. gondii* (Pfefferkorn and Borotz, 1994a; McFadden et al., 2001).

## Mutants of *T. gondii* resistant to artemisinin (ART)

ART is a natural product that is produced by *Artemisia annua* plant. This important compound plays an indispensable role for combating malaria (Cui et al., 2015). ART is also effective against *Toxoplasma in vitro* and *in vivo* (Schultz et al., 2014), although it is not generally used in the treatment of toxoplasmosis. Recent concerns about the development of ART resistance have led to the exploration of its mechanisms of action. Berens et al. (1998) characterized five clonal isolates that showed cross-resistance to the ART derivatives, dihydroartemisinin and artemether in laboratory studies. In a subsequent study, Nagamune et al. (2007) generated chemically derived *T. gondii* mutants that were resistant to growth inhibition by ART *in vitro*. Three ART-resistant mutants were resistant to the induction of protein secretion from micronemes, a calcium-dependent process that is triggered by artemisinin. Based on these findings, calcium homeostasis is involved in the mechanism of ART action against *T. gondii* and other apicomplexan parasites.

## Mutants of *T. gondii* resistant to 1NM-PP1

*T. gondii* CDPK1 (TgCDPK1) was found to be the target of 1NM-PP1, which is a bumped kinase inhibitor (BKIs). CDPK1 contains an atypically small glycine gatekeeper residue, which allows entry of BKIs into the ATP binding domain. Most mammalian kinases have larger gatekeeper residues, e.g., methionine. CDPK1 is involved in microneme secretion and host cell invasion and egress. When TgCDPK1 was mutated at position 128 from glycine to methionine, parasites became BKI resistant (Sugi et al., 2010). Resistance to 1NM-PP1 can also be acquired via a mutation in *T. gondii* mitogen-activated protein kinase like 1, which indicates that this kinase could also be a target (Sugi et al., 2013, 2015). However, CDPK1 has become an important drug target for more recently developed and largely improved BKIs in a variety of apicomplexans beside *T. gondii* (Van Voorhis et al., 2017).

## Mutants of *T. gondii* resistant to dinitroanilines

*T. gondii* is sensitive to dinitroaniline compounds, which disrupt microtubules without affecting host cells. *T. gondii* containing alpha-tubulin point mutations are dinitroaniline resistant. Ma et al. (2008) identified *T. gondii* lines that have suppressed microtubule defects in G142S or F52Y mutations. In addition, secondary resistant mutations were isolated that corrects fitness defects in the *T. gondii* parasite. Based on the current findings, targeting parasite microtubules can be a viable strategy for developing new anti-parasitic therapies.

## Mutants of *T. gondii* resistant to anti-coccidial drugs

Anti-coccidial agents were assessed in *T. gondii* mutants for development of resistance *in vitro*. Mutants had 20- to 50-fold-reduced susceptibility to decoquinate, arprinocid-N-oxide, and CP-25,415. In addition, ionophore-resistant *T. gondii* mutants were explored *in vitro*; however, resistance to all of the mutants except ionophores occurs in coccidia *in vivo*. The availability of a *T. gondii* mutant resistant to a different drug could aid for assessing the risk of developing resistance in Eimeria species (Ricketts and Pfefferkorn, 1993).

Diclazuril, an anticoccidial compound, is a safe and effective drug that inhibits tachyzoite production of RH strain in *T. gondii* by >97% at therapeutic dose levels (Oz, 2014). Lindsay et al. (1995) have shown that formation of *T. gondii* tissue cysts was not prevented by treatment with diclazuril, *in vitro*. They also showed that GT-1, WTD-3 strains, and a mutant RH strain of *T. gondii* were resistant to 1.0 μg/ml of diclazuril.

Monensin is a polyether anti-coccidial antibiotic that has been effective against *T. gondii*. However, within 3 years of the drug introduction, monensin-resistant Eimeria maxima were noted. Thus, *T. gondii* was used for studying the monensin's mechanisms of resistance. The investigators have shown that resistance phenotype is caused by the disruption of *T. gondii* homologs MSH-1 (a homolog of the DNA repair enzyme, MutS). Interestingly, this enzyme localizes to the *T. gondii* parasite mitochondrion (Garrison and Arrizabalaga, 2009). Subsequent studies showed that the disruption of the autophagy pathway could result in drug resistance. Autophagy pathway is a potentially important model of cell death of *T. gondii* in response to monensin (Lavine and Arrizabalaga, 2012).

## Mode of drug action and mechanism of drug resistance in *T. gondii*

Several targets were identified against *T. gondii* including folate synthesis pathway, mitochondrial electron transport chain, calcium dependent ATPases, protein synthesis, mitogen-activated protein kinase 1, enzyme TgDHODH, and microtubules for PYR and SDZ, atovaquone, ART, clindamycin, spiramycin and azithromycin, 1NM-PP1 and 1-Hydroxyquinolones HDQ, and compound B, respectively. Thus, drug resistant mutants are approved tools for the characterization of drug targets for future to select new anti-*Toxoplasma* drugs with specific activity against the parasite.

Also, mechanisms of drug resistance in *T. gondii* have been described. Interestingly, analogous amino acid substitutions in the *Toxoplasma* enzyme have been identified to confer PYR resistance in transfected parasites (Donald and Roos, 1993). Moreover, resistance to clindamycin, spiramycin and azithromycin is encoded in the rRNA genes of the 35-kb genome in *T. gondii*.

There are numerous reports with a focus on identifying SDZ resistance mechanisms. However, *T. gondii* SDZ resistance mechanism has not been proved so far. As a consequence, understanding mechanisms of drug resistance in *T. gondii* is essential for controlling the disease particularly among immunocompromised patients. Also, it helps identify targets that are crucial to the parasite and predicts which combinations of drugs should act synergistically (McFadden et al., 2001).

## Recent trends in drug resistance in *T. gondii*

Studies in the past 10 years indicated that drug resistance to SDZ is actually increased. Most resistant strains were found in clinical cases between 2013 and 2017. However, a possible resistance was reported in three strains of *T. gondii* in 2008. Also, six strains resistant to SDZ were found in clinical cases between 2013 and 2017.

The only worrying trend was a very slight recent increase in SDZ resistance to Brazilian *T. gondii* strains obtained from livestock and humans newborns with congenital toxoplasmosis between 2016 and 2017 where *T. gondii* prevalence in Brazil is high (77.5%) (Pappas et al., 2009). Thus, establishing a more effective therapeutic scheme in the treatment of toxoplasmosis is critically needed.

## Conclusions

Recent experimental studies in clinical cases have clearly shown that drug resistance in *Toxoplasma* is ongoing. The emergence of *T. gondii* strains resistant to current drugs reviewed here represents a concern not only for treatment failure but also for increased clinical severity in immunocompromised patients. Thus, understanding mechanisms of drug resistance is essential for controlling the disease and it helps identify targets that are crucial to the parasite and predicts which combinations of drugs should act synergistically. Also, establishing a more effective therapeutic scheme in the treatment of toxoplasmosis, particularly among high-risk individuals is critically needed. Additionally, monitoring the presence of resistant parasites, particularly in food products, would thus seem a prudent public health measure. Further development of a greater understanding of exact mechanisms of drug resistance in *T. gondii* is needed to improve the therapeutic outcomes in patients.

## Author contributions

AD conceived the study. AD and MS designed the study protocol. MM, SS, and AT searched the databases. MM wrote the manuscript. SM and SA critically revised the manuscript. All authors read and approved the final manuscript for publication.

### Conflict of interest statement

The authors declare that the research was conducted in the absence of any commercial or financial relationships that could be construed as a potential conflict of interest.
